# Phenotype-Preserving Co-culture of Osteoblasts and Chondrocytes Enhances Bone-Cartilage Interface Integration in a PRP-Augmented Scaffold

**DOI:** 10.1007/s13770-025-00727-8

**Published:** 2025-06-16

**Authors:** Sunhyung Lee, Jinwoo Nam, Hong Seok Kim, Jeong Joon Yoo

**Affiliations:** 1 Department of Orthopaedic Surgery, Seoul National University College of Medicine, Seoul, Korea; 2 Department of Orthopaedic Surgery, SMG-SNU Boramae Medical Center, Seoul, Korea; 3 Department of Orthopaedic Surgery, Seoul National University Hospital, 101 Daehak-Ro, Jongno-Gu, Seoul, 03080 Korea

**Keywords:** Scaffold-based tissue engineering, Osteochondral repair, Co-culture of osteoblasts and chondrocytes, PRP

## Abstract

**Background:**

Effective bone-cartilage integration remains a challenge in orthopedic surgery. Conventional methods often fail to reconstruct the native osteochondral interface. This study explores a scaffold-mediated approach utilizing co-cultured osteoblasts and chondrocytes, with platelet-rich plasma (PRP) as a potential promotor for bone-cartilage interface healing.

**Methods:**

We developed a co-culture system integrating both osteoblasts and chondrocytes on PLGA scaffolds, either with or without PRP supplementation. Cell phenotype maintenance was evaluated by RT-PCR, while morphological analysis was performed by scanning electron microscopy and fluorescence microscopy. To assess healing potential, we created a gap-mimic construct comprising bone, scaffold, and cartilage layers, which was implanted subcutaneously in BALB/c-nude mice. Gap healing was evaluated at 4 and 8 weeks through macroscopic examination, quantitative adhesion analysis, and histological assessment of cellular invasion.

**Results:**

Co-cultured osteoblasts and chondrocytes maintained their phenotypes on PLGA scaffolds, with PRP significantly enhancing cell adhesion (215% increase for chondrocytes, 120% for osteoblasts) and proliferation. In vivo, cell-containing scaffolds demonstrated significantly greater attachment at the bone-cartilage interface compared to acellular constructs. PRP-treated scaffolds exhibited higher attachment rates (82.3% vs 76.7%) and cellular invasion (5/6 vs 3/6 constructs) at 8 weeks, with invasion observed as early as 4 weeks in the PRP group, suggesting accelerated remodeling.

**Conclusion:**

This study demonstrates the feasibility of developing transplantable scaffolds containing co-cultured osteoblasts and chondrocytes while preserving their phenotypes. These scaffolds exhibit significant potential in promoting healing at the bone-cartilage interface, with PRP further enhancing proliferation and improving the scaffold’s ability to promote bone-cartilage interface healing.

## Introduction

Effective bone-cartilage integration remains a significant clinical challenge due to limited regenerative capacity. Conditions like osteonecrosis of the femoral head (ONFH), osteochondritis dissecans (OCD), and osteochondral lesion of the talus (OLT) require effective bone-cartilage interface healing to restore joint function and prevent degenerative changes. Despite various treatment strategies, achieving complete osteochondral integration remains a major hurdle in orthopedic surgery.

Several techniques have been employed for bone-cartilage healing. Microfracture [1, 2] induces bone marrow stimulation to recruit progenitor cells for cartilage repair; however, it often results in fibrocartilage formation rather than hyaline cartilage, leading to inferior mechanical properties and durability [3, 4]. Autologous chondrocyte implantation (ACI) [5] allows transplantation of cultured chondrocytes to regenerate cartilage, yet is limited by donor site morbidity, inconsistent chondrocyte phenotype retention, and challenges in subchondral bone integration [4, 6]. Osteochondral autograft or allograft transplantation (OATS) [7] replaces the defect with osteochondral plugs, but graft availability and biomechanical property mismatches remain major concerns [8–10]. In ONFH, the trap-door technique allows curetting necrotic bone tissue and bone grafting before repositioning the cartilage layer, but its efficacy remains suboptimal [11–14]. Moreover, scaffolds incorporating various biomaterials and growth factors have been explored to enhance osteochondral healing. However, these approaches often focus on either cartilage regeneration or bone healing independently, failing to address the biological and mechanical complexity of the bone-cartilage interface comprehensively [15].

While previous studies have investigated different strategies for repair between bone and cartilage, an approach that simultaneously incorporates both osteoblasts and chondrocytes at the bone-cartilage interface has not been systematically explored. Given the critical role of osteoblasts in bone formation and chondrocytes in cartilage maintenance, a co-culture system that facilitates their interaction within a scaffold may offer a promising solution enhancing osteochondral integration.

Furthermore, platelet-rich plasma (PRP), which contains various growth factors that enhance cellular proliferation, extracellular matrix synthesis, and tissue repair, has been proposed as a potential adjunct to optimize scaffold-based regeneration [16–20]. PRP is rich in platelet-derived growth factor (PDGF), transforming growth factor-beta (TGF-β), and vascular endothelial growth factor (VEGF), all of which play crucial roles in tissue repair. Beyond its biological effects, PRP has been shown to modify the surface properties of biomaterials, enhancing cell adhesion, proliferation, and differentiation [21–24]. Through surface modification, PRP facilitates the formation of a bioactive environment that promotes cellular interactions and tissue integration [24]. PRP-treated surfaces have been shown to improve osteoblast attachment [21] and proliferation while supporting chondrocyte viability [23], suggesting its potential in optimizing scaffold-based osteochondral regeneration. Given these properties, PRP is hypothesized to not only support cell proliferation but also facilitate bone-cartilage interface healing by promoting cellular interaction and matrix deposition.

To address the challenges of bone-cartilage interface repair, this study aims to develop a scaffold-mediated strategy that integrates both osteoblasts and chondrocytes in a co-culture system. The study has three primary objectives: (1) to develop a transplantable scaffold containing co-cultured osteoblasts and chondrocytes while ensuring the maintenance of their respective phenotypes; (2) to evaluate whether the cell-containing scaffold could facilitate healing at the bone-cartilage interface, promoting structural and cellular integration; and (3) to investigate the role of PRP in enhancing both cellular proliferation and the scaffold’s regenerative potential at the bone-cartilage junction.

## Materials and methods

### Cell preparation and culture

#### Preparation of human chondrocytes and osteoblasts

Human articular chondrocytes (HCs) were isolated from femoral head cartilage collected during total hip arthroplasty. Informed consent was obtained for all specimens, and the study was approved by the Institutional Review Board (No. H-2107-030-1232). Full-thickness cartilage was minced and digested enzymatically at 37 °C with 0.2% protease (Type XIV; Sigma, St. Louis, MO, USA) for 1 h, followed by 0.2% collagenase (Type IA; Sigma) for 3 h. The resulting suspension was filtered through a 70-μm nylon sieve to remove undigested fragments.

Human osteoblasts (hFOB 1.19, CRL-11372; American Type Culture Collection, Manassas, VA, USA) were cultured in high-glucose Dulbecco’s Modified Eagle’s Medium with nutrient mixture F12 (DMEM/F12; Gibco, Invitrogen, Carlsbad, CA, USA), supplemented with 10% fetal bovine serum (FBS; Gibco) and 1% Penicillin–Streptomycin (Thermo Fisher Scientific, IL, USA). Cells were maintained in a humidified incubator at 37 °C with 5% CO_2_. When cultures reached 70–80% confluency, cells were detached using 0.25% trypsin–EDTA (Gibco) and passaged. Passages 1 and 2 were used for subsequent experiments.

#### Preparation of platelet-rich plasma (PRP)

Allogenic leukocyte-depleted PRP was prepared by collecting peripheral blood from healthy donors into anticoagulant-treated tubes. Blood was centrifuged at 150g for 10 min to separate red blood cells, buffy coat, and plasma. The plasma layer, rich in platelets and depleted of leukocytes, was aspirated while avoiding the buffy coat. A second centrifugation step was performed at a higher speed if necessary to further concentrate the platelets.

### Scaffold-based co-culture

#### Scaffold fabrication, cell labeling, and seeding

Poly-lactic-co-glycolic-acid (PLGA) mesh scaffolds (Ethicon; Johnson & Johnson, USA) measuring 10 × 8 mm, were used. Scaffolds were placed in uncoated 24-well plates, and each scaffold was seeded with 1 × 104 chondrocytes and osteoblasts. Prior to seeding, osteoblasts and chondrocytes were labeled using fluorescent dyes for cell tracking. Osteoblasts were stained with 3,3′-Dioctadecyloxacarbocyanine Perchlorate (DiO, green; Invitrogen), while chondrocytes were labeled with 1,1′-Dioctadecyl-3,3,3′,3′-Tetramethylindocarbocyanine Perchlorate (DiI, red; Invitrogen). After labeling, cells were washed with PBS to remove excess dye before seeding onto the scaffold. To ensure homogeneous cell distribution and prevent cell sedimentation at the bottom of the well, the plates were placed on a bidirectional rotator at 50Hz for 24 h at 37 °C.

#### PRP application and co-culture conditions

Two types of scaffolds were prepared: PRP-containing and PRP-free. For PRP-containing scaffolds, PRP was activated by adding 10% calcium chloride before mixing with the cell suspension. The activated PRP was applied to the scaffold before the co-culture process. Both PRP-containing and PRP-free scaffolds were incubated under the same culture conditions described in Sect. 1.1 to maintain cell viability and proliferation, ensuring efficient cell attachment and growth on the scaffold.

### Cell proliferation, phenotype, and morphological analysis

#### Cell adhesion & proliferation analysis

Cell adhesion and proliferation were assessed using the Cell Counting Kit-8 (CCK-8, Dojindo, Tokyo, Japan), following the manufacturer’s instructions. The cell adhesion assay was performed 24 h post-seeding, where cells were washed with DPBS (GibcoBRL), and 10 μL of CCK-8 solution per 100 μL of media was added. The absorbance was measured at 450 nm using a microplate reader (Spectramax M5, Versamax; Molecular Devices) after 3 h of incubation to form formazan crystals. Cell proliferation was subsequently evaluated at 3- and 7-days post-seeding using the same method.

#### Phenotype analysis (RT-PCR)

To confirm phenotype maintenance during co-culture, RT-PCR was performed at 24 h, 3 days, and 7 days post-seeding. Total RNA was extracted using the RNeasy Mini Kit (Qiagen, Hilden, Germany), and cDNA synthesis was performed using the Transcriptor First Strand cDNA Synthesis Kit (Roche, Basel, Switzerland). Primers targeting collagen II and aggrecan for chondrocytes, and alkaline phosphatase (ALP) and osteocalcin (OC) for osteoblasts were used, with GAPDH as an internal control. PCR amplification was conducted with an initial denaturation at 95 °C for 3 min, followed by 28–32 cycles of denaturation at 95 °C for 15 s, annealing at 55 to 68 °C, and extension at 72 °C for 30 s depending on the target gene. Table 1 provides detailed primer sequences along with the specific annealing temperatures and PCR cycles for each gene.

**Table 1:** Primer sequences and PCR conditions for RT-PCR analysis

Target transcript	Primer sequence	Annealing temperature (°C)	PCR cycles	Predicted size (bp)
Collagen II	(F) 5′-GAATTCGGTGTGGACATAGG-3′ (R) 5′-TACAGAGGTGTTTGACACAG-3′	60	28	428
Aggrecan	(F) 5′-TACTCTGGGTTTTCGTGACTC-3′ (R) 5′-CGATGCCTTTCACCACGACTT-3′	60	32	450
ALP	(F) 5′-TGGAGCTTCAGAAGCTCAACACCA-3′ (R) 5′-ATCTCGTTGTCTGAGTACCAGTCC-3′	61	28	454
OC	(F) 5′-ATGAGAGCCCTCACACTCCTCGCCCTA-3′ (R) 5′-GACCGGGCCGTAGAAGCGCCGATAGG-3′	68	28	300
GAPDH	(F) 5′-ATTGTTGCCATCAATGACCC-3′ (R) 5′-AGTAGAGGCAGGGATGATGTT-3′	55	28	546

F: forward, R: reverse, ALP: alkaline phosphatase, OC: osteocalcin, GAPDH: glyceraldehyde-3-phosphate dehydrogenase

#### Morphological analysis (FE-SEM & fluorescence microscopy)

The morphology and spatial distribution of co-cultured chondrocytes and osteoblasts on the PLGA mesh scaffolds were evaluated using field emission scanning electron microscopy (FE-SEM) and fluorescence microscopy.

For FE-SEM analysis, cells on the scaffolds were washed three times with PBS and fixed in 2% glutaraldehyde (Sigma) at 4 °C for 2 h. After fixation, samples were dehydrated in increasing concentrations of ethanol (70 to 100%) and dried in a vacuum. The dried samples were then sputter-coated with a thin gold–palladium layer and imaged using an FE-SEM (JSM-740F, JEOL Ltd., Japan) at 24 h, 3 days, and 7 days post-seeding.

Fluorescence imaging was conducted using a DMi8 inverted fluorescence microscope (Leica Microsystems, Germany) to evaluate the spatial distribution and integration of labeled chondrocytes and osteoblasts on the scaffold. Images were captured at 24 h, 3 days, and 7 days post-seeding to monitor cell localization and potential migration patterns over time.

### Gap-mimic construct and in vivo implantation

#### Preparation of the gap-mimic construct

**Fig. 1:**
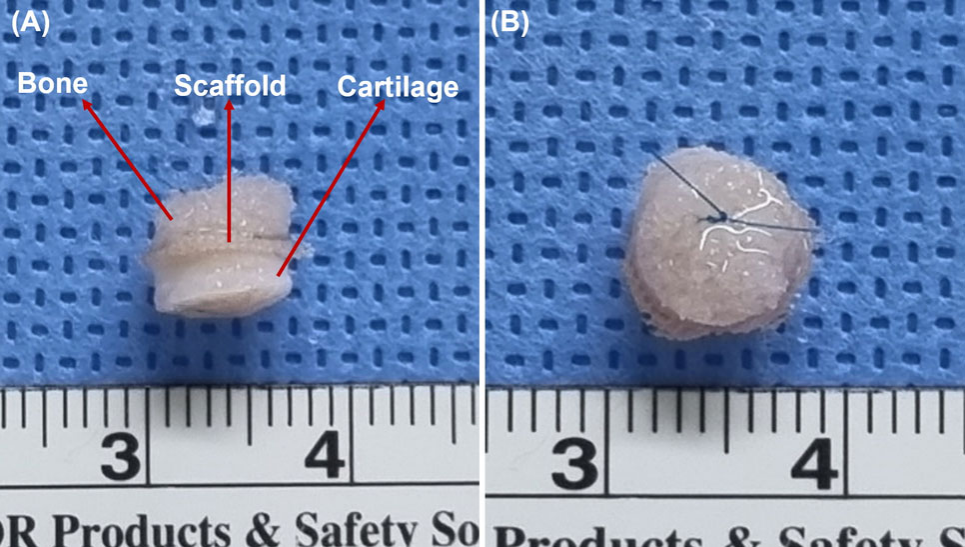
Preparation of the gap-mimic construct. **A** Gap-mimic construct composed of cartilage, scaffold, and bone. **B** Final assembled construct secured with 6–0 nylon sutures for in vivo implantation

We produced a gap-mimic construct comprising three layers: cartilage, scaffold, and bone (Fig. 1A). Articular cartilage and bone were harvested from the resected femoral head during total hip arthroplasty. Articular cartilage underwent more than three freeze–thaw cycles at -70 °C to eliminate resident cells, ensuring that only the scaffold-seeded cells contribute to the observed effects. Bone tissue was subjected to the same freeze–thaw cycles as cartilage, followed by sterilization via irradiation at 25 kGy to ensure complete devitalization while maintaining the extracellular matrix structure, with all procedures performed at our institution's bone bank. Bone and cartilage tissues were processed into disc-shaped constructs with a diameter of 6 mm.

The PLGA scaffold was placed between the bone and cartilage discs and sutured using 6–0 nylon (Fig. 1B). Four experimental groups were prepared: (1) scaffold alone, (2) scaffold with co-cultured cells, (3) scaffold with PRP alone, and (4) scaffold with PRP and co-cultured cells.

#### In vivo implantation

Twenty-four 6-week-old BALB/c-nude mice were used in this study, with approval from the Institutional Animal Care and Use Committee (IACUC approval No. 22-0219-S1A0). Mice were anesthetized with 2–3% isoflurane for induction and 1.5–2.5% for maintenance. Constructs were implanted subcutaneously on both sides of the dorsal skin. Mice were divided into two groups described above based on PRP treatment, each consisting of 12 mice. At 4 and 8 weeks post-implantation, twelve mice were euthanized for construct retrieval and analysis.

### Evaluation of gap healing in vivo

**Fig. 2:**
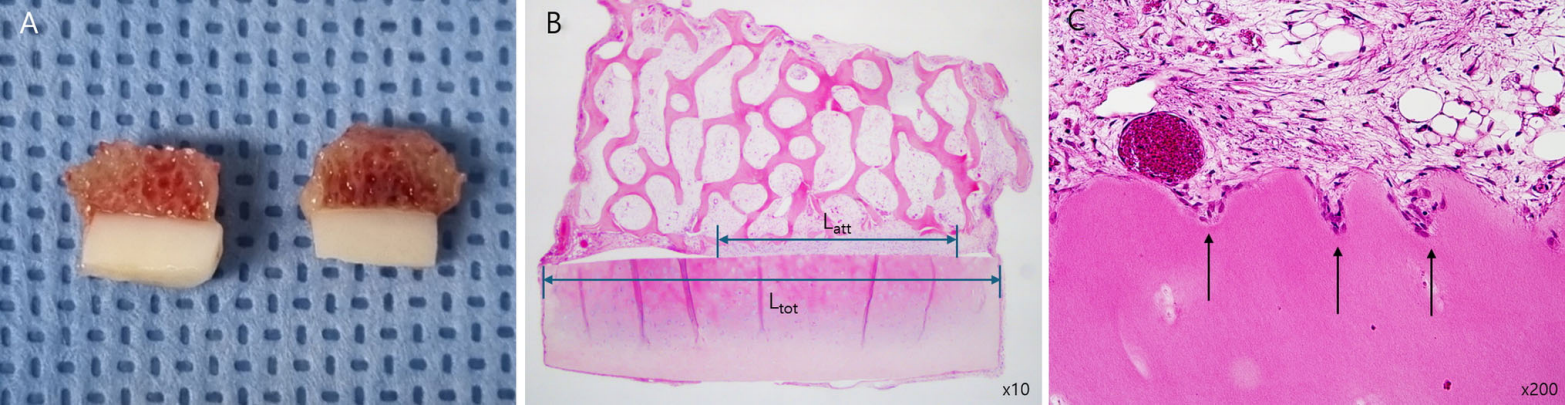
Evaluation of gap healing at the bone-cartilage interface. **A** Macroscopic analysis of bisected constructs assessing gross bonding at the bone-cartilage interface. **B** Quantitative analysis of attachment, where the total construct diameter L_tot_ is 6 mm. The degree of attachment was measured as the percentage of the attached length L_att_ relative to L_tot_, using a 10 × magnification microscope. **C** Histological analysis (H&E staining, × 200) showing bone-cartilage interdigitation. The image represents a case with evident cellular invasion (arrow) at the interface

To evaluate gap healing, three assessments were conducted: macroscopic analysis to examine gross structural bonding, quantitative analysis to measure the degree of attachment, and histological analysis to assess qualitative features of cellular invasion at the bone-cartilage interface (Fig. 2).

Gap healing was first evaluated through macroscopic analysis (Fig. 2A). After removing the nylon suture, the retrieved constructs were bisected vertically, and the exposed bone-cartilage interface was examined macroscopically to assess the gross bonding. After macroscopic analysis, samples underwent decalcification to facilitate histological processing. Decalcification was performed using 10% EDTA (pH 7.4) at room temperature, with the solution changed every 48 h for 2–3 weeks until complete softening was achieved. The bisected samples were then fixed in 10% phosphate-buffered formalin, embedded in paraffin, sectioned at 5 μm thickness, and stained with hematoxylin and eosin (H&E) for histological examination.

Quantitative analysis was performed to evaluate the degree of attachment (Fig. 2B). The degree of attachment was measured using a 10 × magnification microscope, with the contact length between the cartilage and bone along the bisected 6 mm diameter of the construct quantified. The percentage of the attached length relative to the total diameter was then calculated, providing a quantitative assessment of the physical integration between the bone and cartilage.

Lastly, histological analysis was performed to assess cellular invasion, focusing on bone-cartilage interdigitation as a key indicator of active healing (Fig. 2C). Cellular invasion was considered present if the interface exhibited a serpentine pattern, with cells from the bone or cartilage layer infiltrating the opposing layer. To complement this, fluorescence microscopy was performed using DiO (green, osteoblasts) and DiI (red, chondrocytes) to evaluate the localization and distribution of co-cultured cells within the construct.

### Statistical analysis

The degree of attachment, a continuous variable, and the presence of cellular invasion, a categorical variable, were analyzed to determine if there were statistically significant differences between constructs with and without cells in the PRP (−) and PRP (+) groups. Due to the small sample size, which precludes the assumption of normal distribution, the Mann–Whitney U test was used to compare the degree of attachment (continuous data), while the Chi-square test or Fisher's exact test was applied to evaluate the presence of cellular invasion (categorical data). Additionally, statistical analyses were conducted to assess differences in attachment and invasion between the 4-week and 8-week time points within the PRP (−) and PRP (+) groups.

## Results

### Co-culture process

**Fig. 3:**
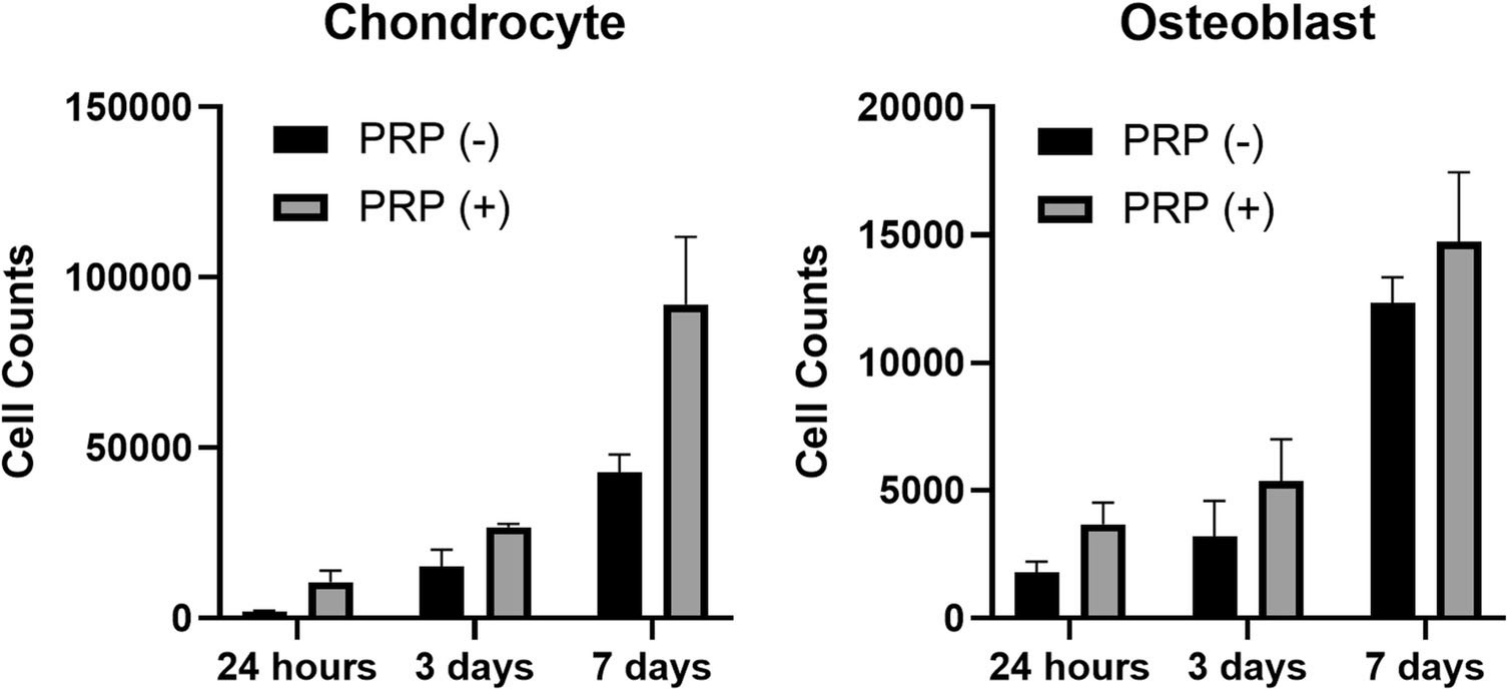
Cell adhesion and proliferation analysis. Cell counts of chondrocytes (left) and osteoblasts (right) on poly-lactic-co-glycolic-acid (PLGA) scaffolds with and without platelet-rich plasma (PRP) at 24 h, 3 days, and 7 days post-seeding. PRP enhanced initial adhesion in both cell types and increased chondrocyte proliferation by 115% and osteoblast proliferation by 20% at day 7 compared to the PRP-free group. Data are presented as mean ± standard deviation

**Fig. 4:**
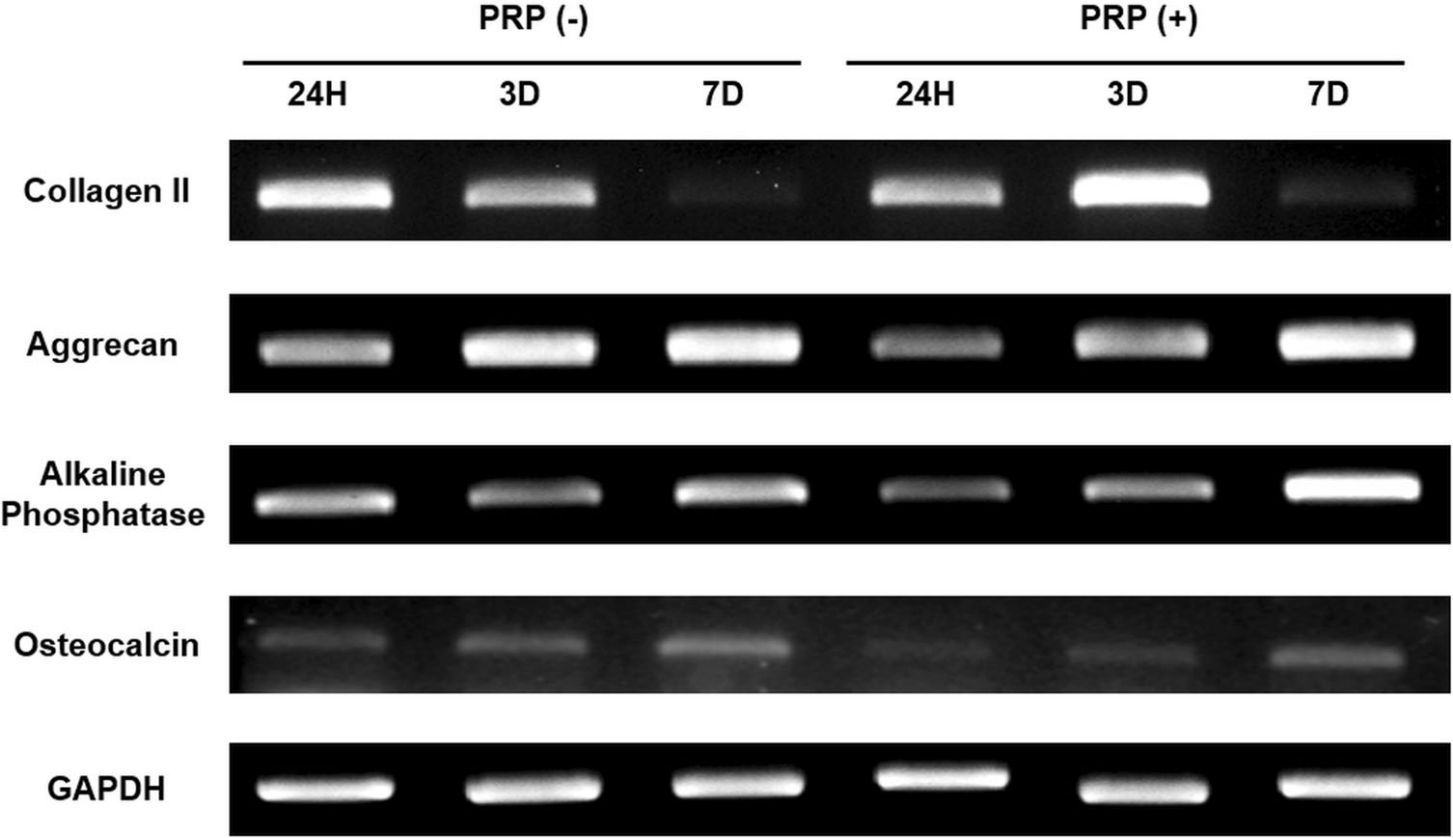
RT-PCR analysis of chondrocyte and osteoblast markers. Gene expression of collagen II, aggrecan, alkaline phosphatase (ALP), and osteocalcin in co-cultured cells with and without platelet-rich plasma (PRP) at 24 h, 3 days, and 7 days. PRP enhanced collagen II expression at day 3 and ALP expression at days 3 and 7. Aggrecan increased over time, while osteocalcin remained weakly expressed. GAPDH served as an internal control

**Fig. 5:**
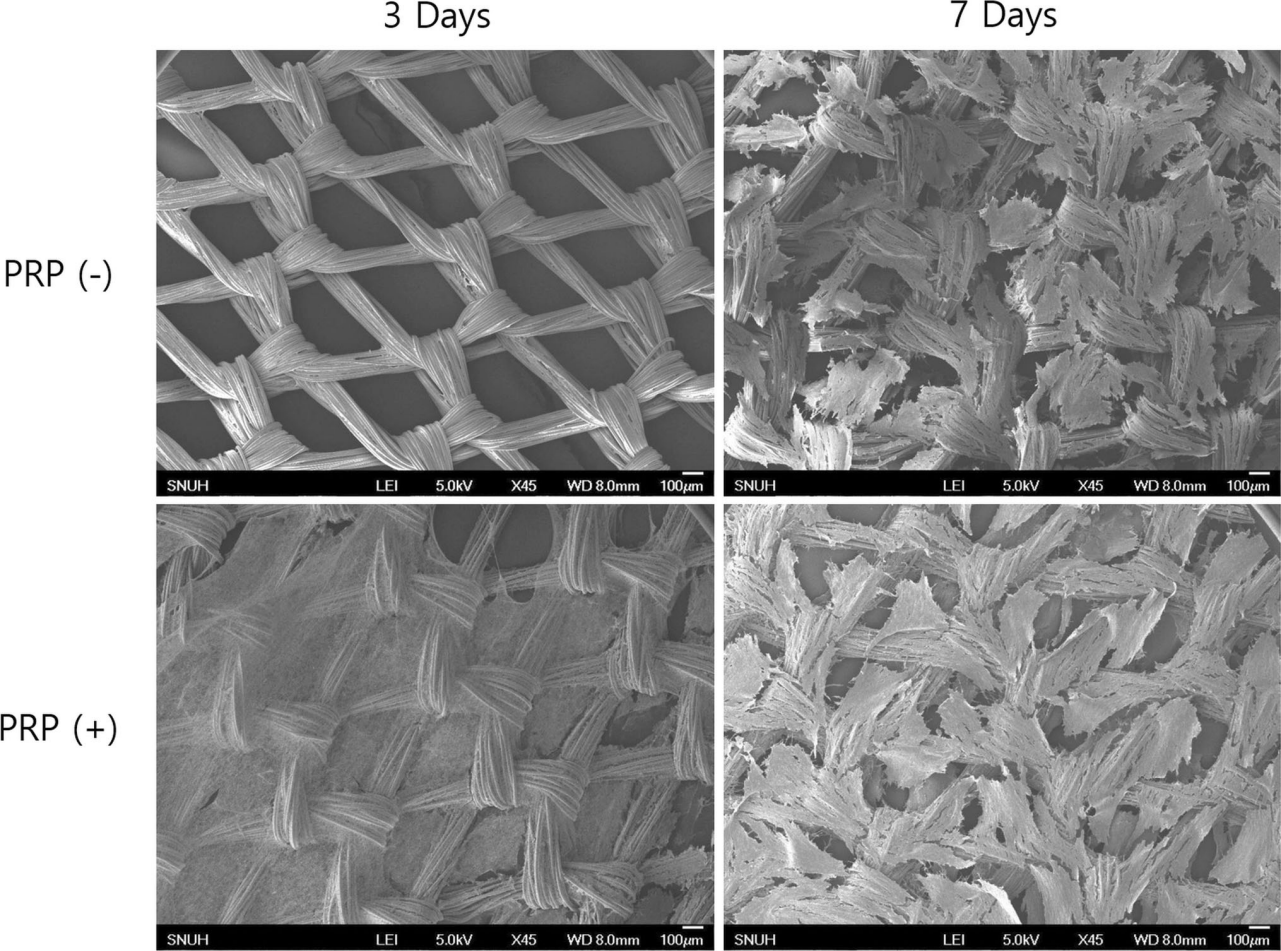
Field emission scanning electron microscopy (FE-SEM) images of poly-lactic-co-glycolic-acid (PLGA) scaffolds. At day 3, platelet-rich plasma (PRP) (−) scaffolds exhibit minimal cell adhesion, while PRP (+) scaffolds show greater cellular attachment. By day 7, both groups display increased cell coverage, with the PRP (+) group forming denser and more organized cellular distribution. Scale bar = 100 μm

**Fig. 6:**
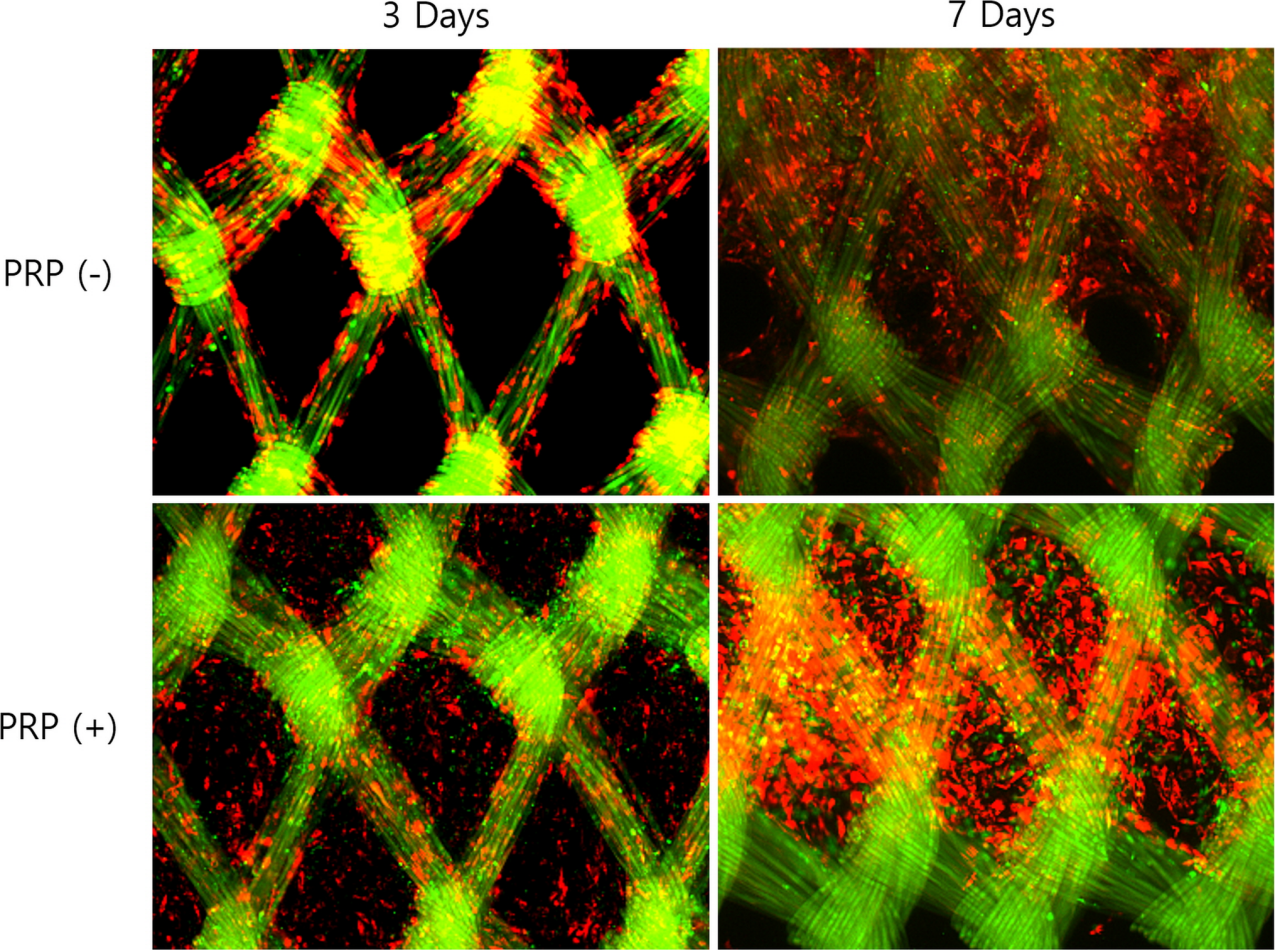
Fluorescence images of chondrocytes (DiI, red) and osteoblasts (DiO, green) on poly-lactic-co-glycolic-acid (PLGA) scaffolds. At day 3, platelet-rich plasma (PRP) (+) scaffolds exhibit greater initial cell attachment compared to PRP (−) scaffolds. By day 7, both groups show increased cell coverage, with PRP (+) scaffolds demonstrating a higher density and more uniform distribution. Osteoblasts are labeled in green (DiO), and chondrocytes in red (DiI)

Figures 3, 4, 5, 6 illustrate the successful adhesion and proliferation of chondrocytes and osteoblasts on the PLGA mesh scaffold while maintaining their respective phenotypes.

#### Cell adhesion and proliferation

Figure 3 shows that PRP enhanced both initial cell adhesion and subsequent proliferation, particularly for chondrocytes. In the PRP-free group, cell counting at 24 h post-seeding revealed that approximately one-fifth of the seeded chondrocytes (1.9 × 103 ± 0.4 × 103 cells, 19% of seeded cells) and osteoblasts (1.8 × 103 ± 0.4 × 103 cells, 18% of seeded cells) successfully adhered to the scaffold. By day 7, chondrocytes exhibited a 23-fold increase relative to adhered cells (42.8 × 103 ± 5.3 × 103 cells), whereas osteoblasts increased approximately sevenfold (12.3 × 103 ± 1.0 × 103 cells).

The PRP-containing group demonstrated enhanced initial adhesion. At 24 h post-seeding, 10.7 × 103 ± 3.2 × 103 chondrocytes (107% of seeded cells) and 3.7 × 103 ± 0.9 × 103 osteoblasts (37% of seeded cells) adhered to the scaffold. By day 7, chondrocytes exhibited an 8.6-fold increase relative to adhered cells (92.1 × 103 ± 19.8 × 103 cells), whereas osteoblasts increased fourfold (14.7 × 103 ± 2.7 × 103 cells). Compared to the PRP-free group, PRP treatment resulted in a 115% increase in chondrocyte proliferation and a 20% increase in osteoblast proliferation, highlighting its positive impact on cell expansion.

#### Phenotype analysis (RT-PCR)

RT-PCR analysis (Fig. 4) confirmed that co-culturing chondrocytes and osteoblasts, along with PRP supplementation, did not alter the expression of their respective phenotypic markers. GAPDH was consistently expressed across all conditions as an internal control.

For chondrocytes, collagen II expression was maintained until day 3 but showed a noticeable decrease by day 7, suggesting potential phenotypic instability over prolonged culture. In contrast, aggrecan expression increased over time, indicating sustained extracellular matrix synthesis and chondrocyte activity. Notably, in the PRP-containing group, collagen II expression was stronger at day 3 compared to the PRP-free group.

For osteoblasts, ALP expression progressively increased throughout the culture period, while osteocalcin expression, though consistently detected, remained weak throughout the culture period. In the PRP-containing group, ALP expression appeared stronger on day 3 and 7 compared to the PRP-free group.

#### Morphological analysis (FE-SEM & fluorescence microscopy)

FE-SEM and fluorescence microscopy analyses collectively demonstrate the progressive increase in cell coverage and spatial distribution on PLGA scaffolds over time, with PRP promoting more extensive and structured cellular organization (Figs. 5, 6).

FE-SEM images reveal that at day 3, PRP-free scaffolds exhibited sparse cellular adhesion, with cells primarily localized around the mesh fibers. In contrast, PRP-containing scaffolds demonstrated a denser cellular distribution covering the mesh structure. By day 7, both groups showed increased cell proliferation, with the PRP-containing group forming a more confluent and structured cellular network, further supporting PRP’s role in enhancing scaffold surface coverage and potential cellular integration (Fig. 5).

Fluorescence microscopy images provided additional information regarding the spatial distribution of cells on the scaffold. At day 3, chondrocytes (DiI-labeled, red) and osteoblasts (DiO-labeled, green) were sparsely distributed across the PRP-free scaffolds, whereas PRP-containing scaffolds showed a higher density of both cell types. By day 7, fluorescence images indicated that cells became more evenly distributed and occupied a greater portion of the scaffold’s porous regions, aligning along the mesh structure (Fig. 6).

### Gap healing potential

#### Macroscopic analysis

Gross bonding at the bone-cartilage interface was consistently observed in all cell-containing constructs, while PRP also facilitated bonding in some acellular constructs. Table 2 shows that at 4 weeks post-implantation, all constructs in both PRP (−) Cell (+) and PRP (+) Cell (+) groups exhibited gross bonding. In contrast, bonding was observed in only 1 out of 6 constructs in the PRP (−) Cell (−) group and 3 out of 6 constructs in the PRP (+) Cell (−) group. By 8 weeks, all PRP (−) Cell (+) and PRP (+) Cell (+) constructs maintained gross bonding. Meanwhile, the PRP (−) Cell (−) group still exhibited bonding in only 1 out of 6 constructs, whereas the PRP (+) Cell (−) group showed increased bonding in 5 out of 6 constructs.

**Table 2:** Macroscopic and histologic analysis of gap healing

		4 Weeks post-implantation				8 Weeks post-implantation			
		Mouse	Gross Bonding	Attachment (%)	Cellular Invasion	Mouse	Gross Bonding	Attachment (%)	Cellular Invasion
PRP (−)	Cell (−)	1	−	0	−	1	−	0	−
		2	+	9	−	2	−	0	−
		3	−	0	−	3	+	59	−
		4	−	0	−	4	−	0	−
		5	−	0	−	5	−	0	−
		6	−	0	−	6	−	0	−
		Total	1/6	1.5 ± 3.7*	0/6	Total	1/6	9.8 ± 24.1*	0/6
	Cell (+)	1	+	79	−	1	+	49	−
		2	+	17	−	2	+	90	+
		3	+	31	−	3	+	91	−
		4	+	84	−	4	+	57	−
		5	+	18	−	5	+	98	+
		6	+	86	−	6	+	75	+
		Total	6/6	52.5 ± 33.9*	0/6	Total	6/6	76.7 ± 20.0*	3/6
PRP (+)	Cell (−)	1	−	0	−	1	+	6	−
		2	−	0	−	2	−	0	−
		3	+	50	−	3	+	55	−
		4	+	18	−	4	+	16	−
		5	+	10	−	5	+	8	−
		6	−	0	−	6	+	71	−
		Total	3/6	13.0 ± 19.5*	0/6	Total	5/6	26.0 ± 29.6*	0/6
	Cell (+)	1	+	79	−	1	+	88	+
		2	+	92	+	2	+	100	+
		3	+	88	−	3	+	97	+
		4	+	14	−	4	+	36	

#### Quantitative analysis

**Fig. 7:**
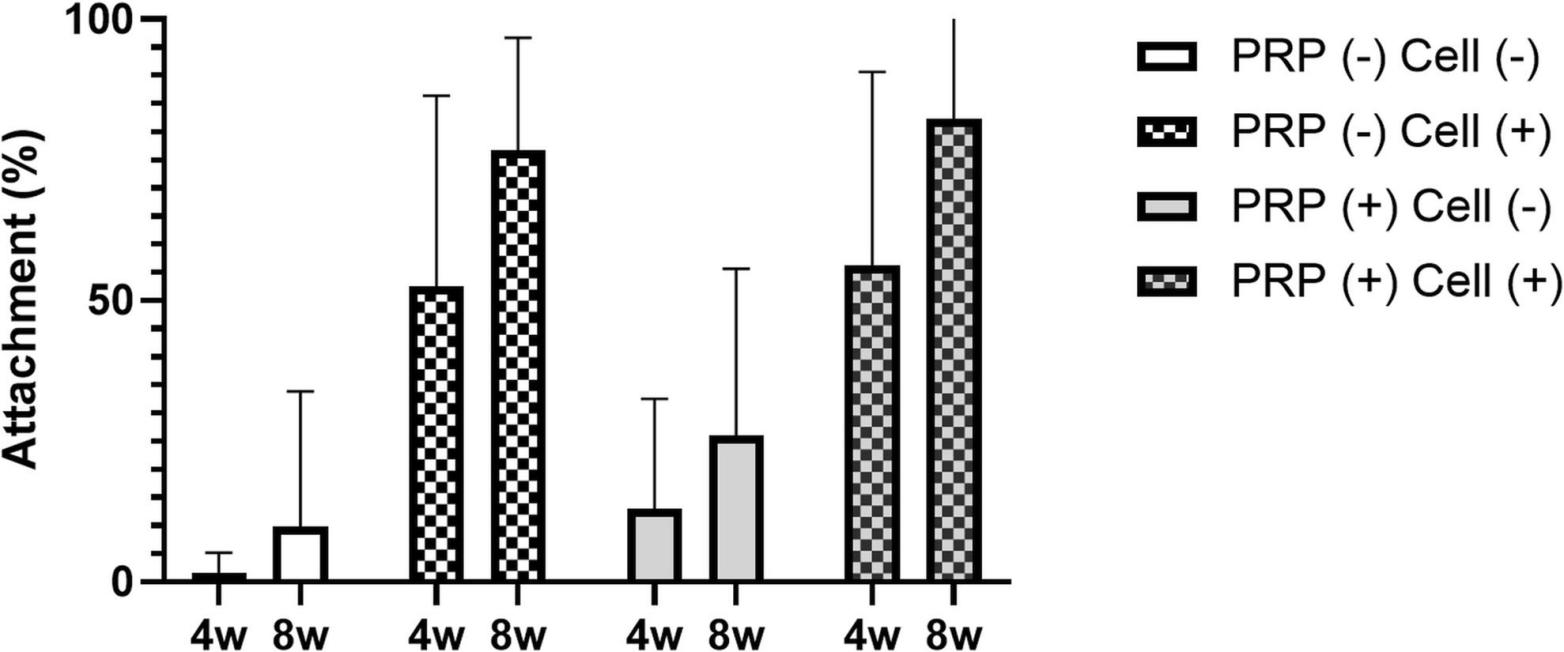
Quantitative analysis of attachment at the bone-cartilage interface. Attachment (%) was measured in platelet-rich plasma (PRP) (−) and PRP (+) groups with and without cells. Constructs seeded with cells exhibited significantly higher attachment compared to acellular groups. PRP (+) constructs demonstrated a trend toward increased attachment over time. Data are presented as mean ± standard deviation

In both groups, cell-seeded constructs exhibited significantly greater attachment compared to acellular constructs (*p* = 0.01) (Table 2, Fig. 7). At 8 weeks post-implantation the PRP (+) Cell (+) group demonstrated an average attachment rate of 82.3%, while the PRP (−) Cell (+) group exhibited an average attachment of 76.7%. While the PRP-containing group showed a higher attachment rate, no statistically significant difference was observed between the two cell-seeded groups at 8 weeks (*p* = 0.67) or at 4 weeks (*p* = 0.70).

Over time, the degree of attachment increased in both PRP-treated and PRP-free groups. Although the PRP (−) group did not exhibit a substantial difference in attachment between 4 and 8 weeks (*p* = 0.163), and the PRP (+) group showed a similar pattern (*p* = 0.127).

#### Histological and fluorescence analysis

**Fig. 8:**
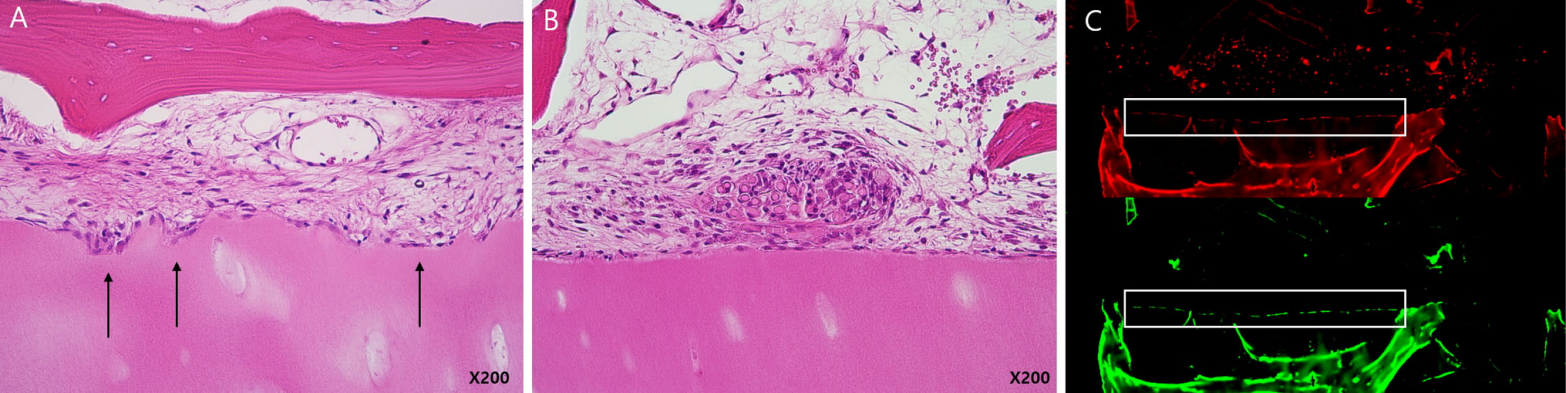
Histological and fluorescence analysis of the bone-cartilage interface. **A** Representative hematoxylin and eosin (H&E) stained image showing a serpentine bone-cartilage interface with evident cellular invasion (arrow), indicating active remodeling and integration between bone and cartilage. (× 200 magnification) **B** H&E stained image displaying a flat bone-cartilage interface with no apparent cellular invasion. The two tissues appear attached but without evident interdigitation, suggesting limited biological interaction. (× 200 magnification) **C** Fluorescence microscopy images of osteoblasts (green, DiO-labeled) and chondrocytes (red, DiI-labeled) showing their localization within the scaffold. The white boxes indicate the bone-cartilage interface, highlighting the spatial distribution of the co-cultured cells within the construct

Cellular invasion was observed exclusively in cell-seeded constructs, with a higher incidence in PRP-treated groups (Table 2) (Fig. 8A, B). At 8 weeks post-implantation, invasion was detected in 3 out of 6 constructs in the PRP (−) Cell (+) group and in 5 out of 6 constructs in the PRP (+) Cell (+) group, suggesting a trend toward increased invasion in the presence of PRP.

While PRP-containing constructs exhibited higher invasion rates compared to PRP-free constructs (5 out of 6 vs. 3 out of 6), the difference did not reach statistical significance (*p* = 0.55). Similarly, when comparing invasion trends over time, both PRP (−) and PRP (+) groups exhibited higher cellular invasion at 8 weeks compared to 4 weeks, but the differences were not statistically significant (*p* = 0.182 and *p* = 0.08, respectively).

Fluorescence microscopy further confirmed the localization and distribution of co-cultured cells within the scaffold region at the bone-cartilage interface (Fig. 8C). DiO-labeled osteoblasts (green) and DiI-labeled chondrocytes (red) were observed in the scaffold area in all cell-seeded constructs, regardless of PRP presence.

## Discussion

This study successfully demonstrated the feasibility of co-culturing chondrocytes and osteoblasts on a PLGA scaffold while maintaining their respective phenotypes. These cell-seeded scaffolds enhanced healing at the bone-cartilage interface. The presence of PRP significantly increased cell adhesion, proliferation and scaffold coverage, with a trend suggesting improved attachment and cellular invasion at the bone-cartilage interface. Fluorescence microscopy confirmed that both osteoblasts and chondrocytes remained their specific location within the scaffold at the bone-cartilage interface in all groups, providing evidence for their active role in mediating the integration progress.

Our results confirmed the potential to develop a transplantable scaffold with co-cultured osteoblasts and chondrocytes while maintaining their phenotypic stability. Both cell types exhibited robust proliferation, with chondrocytes increasing 9- to 23-fold and osteoblasts 4- to sevenfold relative to initially adhered cells. RT-PCR analysis confirmed stable expression of chondrogenic and osteogenic markers, with collagen II expression maintained until day 3 and subsequently declining by day 7. In contrast, aggrecan expression increased steadily over time, suggesting sustained extracellular matrix production and metabolic activity of chondrocytes. Among osteogenic markers, ALP expression showed a progressive increase, while osteocalcin remained weakly expressed throughout the culture period. These findings underscore the importance of optimizing culture period prior to in vivo application.

The temporal decline in collagen II expression may raise concerns about chondrocyte phenotype stability; however, we interpret this as a sign of phenotypic drift rather than full dedifferentiation. This interpretation is supported by the concomitant increase in aggrecan, a key marker of cartilage matrix synthesis, which suggests that the chondrocytes remained metabolically active. This drift may be attributed to reciprocal interactions with osteoblasts or intrinsic limitations of scaffold-based culture conditions. Co-culturing osteoblasts and chondrocytes facilitates cellular interactions through paracrine signaling and direct cell contact [25–28]. Osteoblasts modulate chondrocyte proliferation and differentiation by secreting soluble factors that regulate chondrogenic activity [27]. These mechanisms could contribute to the observed modulation of gene expression over time. Prior studies [25, 28] indicate that co-culture can alter differentiation pathways, occasionally leading to phenotype drift [26] or even dedifferentiation [28] under prolonged or suboptimal conditions.

The reduction in chondrocyte marker expression may also stem from intrinsic cellular behavior. Studies [29, 30] have demonstrated chondrocytes exhibit superior differentiation and phenotype stability in a three-dimensional culture system compared to conventional two-dimensional conditions. The decline in collagen II expression may reflect a gradual loss of chondrocyte phenotype in vitro, likely due to inability of the scaffold-based culture system to fully replicating native conditions. These findings emphasize the need to further optimize scaffold composition and culture parameters to better support long-term chondrocyte function and phenotype retention in future studies.

For osteoblast markers, osteocalcin expression remained weak throughout, which aligns with its role as a late-stage differentiation marker [31–33]. Osteocalcin expression is typically upregulated during the mineralization phase [33], which occurs later than the time points examined in this study. Therefore, the relatively weak osteocalcin expression likely reflects the early- to mid-stage differentiation status of the seeded osteoblasts, rather than impaired function.

Cell-containing scaffolds effectively facilitated attachment and cellular invasion at the bone-cartilage interface, as evidenced by microscopic analysis and fluorescence imaging. Cell-seeded constructs exhibited significantly improved attachment compared to acellular scaffolds. The structural remodeling at the bone-cartilage interface, characterized by a transition from flat to serpentine morphology with interdigitation, was observed exclusively in cell-seeded constructs. This interdigitation suggests that cellular activity actively contributed to the integration process, rather than passive physical contact between bone and cartilage. Cellular invasion was predominantly observed at 8 weeks, with 3 cases in the PRP (−) group and 5 cases in the PRP (+) group, although one case was detected as early as 4 weeks in the PRP (+) group. Given that bone healing typically progresses over four to eight weeks post-injury [34], the observed cellular invasion at eight weeks aligns with established bone repair mechanisms. Furthermore, fluorescence microscopy confirmed the localization of osteoblasts and chondrocytes at the bone-cartilage interface, supporting their active role in mediating attachment or cellular invasion.

Our results demonstrated that PRP significantly enhanced initial cell adhesion and proliferation, with chondrocytes exhibiting a 215% increase and osteoblasts a 120% increase compared to PRP-free conditions (Fig. 3), findings that align with previous studies [35, 36]. Additionally, PRP-treated scaffolds exhibited a more extensive cellular network by day 7 (Fig. 5), indicating PRP supports scaffold colonization and extracellular matrix deposition. Despite its proliferative effects, PRP did not disrupt the phenotypic expression of either cell type. Notably, collagen II expression was stronger at day 3 in the PRP-treated group, suggesting that PRP may support early stabilization of the chondrocyte phenotype. Similarly, ALP expression was higher in PRP-treated osteoblasts at days 3 and 7, indicating that PRP may promote osteoblast differentiation without interfering with normal maturation process.

PRP also contributed to gross bonding at the interface, even in the absence of cells. While constructs without cells generally failed to achieve gross bonding, a subset of PRP(+) Cell(−) constructs exhibited attachment, suggesting that PRP may promote direct adhesion between bone and cartilage. Previous studies [37, 38] have proposed that platelet-derived fibrin enhances cartilage defect healing by forming a transient matrix that facilitates tissue integration.

In quantitative analysis, PRP-treated constructs demonstrated a higher attachment rate (82.3% vs. 76.7%, *p* = 0.67) with increased attachment between 4 and 8 weeks in both groups, and the increase was slightly greater in the PRP group (*p* = 0.13). Histological analysis revealed that PRP-treated constructs exhibited greater cellular invasion (5/6 vs. 3/6, *p* = 0.55) and developed more extensive interdigitation patterns at 8 weeks. Notably, cellular invasion was observed as early as 4 weeks in one case of the PRP (+) group, whereas invasion was detected exclusively at 8 weeks in the PRP (−) group. These temporal differences suggest that PRP accelerates the remodeling process at the bone-cartilage interface, facilitating earlier cellular migration and integration. Additionally, fluorescence microscopy confirmed that PRP did not interfere with the distribution of co-cultured cells, as both osteoblasts and chondrocytes maintained their proper localization within the scaffold at the bone-cartilage interface.

While these findings highlight the potential of PRP to enhance both early cell behavior and long-term tissue integration, we acknowledge that the specific active components responsible for these effects were not directly identified in this study. Our aim was to evaluate the overall biological efficacy of PRP as a clinically relevant adjunct. Nonetheless, future studies employing fractionated PRP or targeted inhibition of individual growth factors such as PDGF, TGF-β, and VEGF will be essential to delineate the specific molecular pathways involved in osteochondral interface healing.

Despite these promising findings, this study has several limitations. First, the relatively small sample size may limit the generalizability of the results and likely reduced the statistical power of the analyses. Although PRP showed trends toward enhanced attachment and interdigitation between bone and cartilage, these improvements did not reach statistical significance, possibly due to the limited sample size. Second, our experimental model lacked the complex mechanical loading conditions present in functional joints, making it less representative of clinical scenarios where bone-cartilage interface healing is required. Future studies using larger sample sizes and joint-relevant implantation models will be necessary to validate the scaffold’s efficacy under more clinically realistic conditions. Additionally, future clinical application of these scaffolds necessitates overcoming significant surgical challenges, as precisely positioning them at the bone-cartilage interface within a joint space while minimizing damage to surrounding tissues.

In conclusion, this study demonstrates the feasibility of developing transplantable scaffolds containing co-cultured osteoblasts and chondrocytes while preserving their phenotypes. These scaffolds exhibit significant potential for promoting healing at the bone-cartilage interface, with PRP further enhancing proliferation and improving the scaffold’s ability to support bone-cartilage interface healing.

## Data Availability

The datasets used and/or analyzed during the current study are available from the corresponding author on reasonable request.
